# Benign *struma ovarii* mimicking ovarian cancer in an afghan woman: an unusual case report and literature review

**DOI:** 10.1093/omcr/omag124

**Published:** 2026-07-12

**Authors:** Maryam Yousefi, Elham Sadat Banimostafavi, Mahdiieh Ghoddoosi

**Affiliations:** Department of Obstetrics and Gynecology, School of Medicine, Clinical Research Development Unit, Shahid Beheshti Hospital, Qom university of Medical Sciences, Qom, Iran; Department of Radiology, Gastroenterology and Hepatology Diseases Research Center, Interdisciplinary Research Committee, Shahid Beheshti Hospital, Qom University of Medical Sciences, Qom, Iran; Department of Pathology, Kamkar-Arabnia Hospital, Qom University of Medical Sciences, Qom, Iran

**Keywords:** Struma Ovarii, ovarian teratoma, ovarian cancer, CA-125, benign ovarian tumor, Afghanistan

## Abstract

**Introduction:**

*Struma ovarii* is a rare monodermal teratoma composed predominantly of thyroid tissue. While typically benign, its clinical and radiological presentation can be highly deceptive, often mimicking a malignant ovarian tumor.

**Case Presentation:**

A 52-year-old postmenopausal Afghan woman presented with progressive abdominal distension, pelvic pain, severe ascites, and a complex, highly vascular right adnexal mass. Imaging (ORADS 5) and markedly elevated CA-125 suggested advanced ovarian carcinoma. Despite receiving seven cycles of carboplatin/paclitaxel chemotherapy for presumed primary peritoneal carcinoma, the mass and ascites persisted. Following laparotomy and left salpingo-oophorectomy, histopathology confirmed benign *struma ovarii* without peritoneal carcinomatosis. Postoperatively, ascites resolved and CA-125 normalized. The patient remained well with no recurrence at 14-month follow-up.

**Conclusion:**

This case underscores the critical importance of considering struma ovarii in the differential diagnosis of a complex ovarian mass, even when clinical features and tumor markers strongly suggest malignancy.

## Introduction

*Struma ovarii*, is a specialized form of a mature cystic teratoma, defined as an ovarian teratoma composed predominantly (>50%) of thyroid tissue [1]. It accounts for approximately 1%–3% of all ovarian teratomas and only 0.3%–1% of all ovarian tumors [2]. The clinical presentation is often non-specific, including pelvic pain, a palpable mass, or abdominal distension. In about 5%–10% of cases, it can be associated with ascites and, rarely, with pseudo-Meigs’ syndrome (ascites and hydrothorax) [3].

The principal clinical challenge lies in its potential to mimic ovarian malignancy. Preoperative imaging frequently reveals complex, solid-cystic lesions with vascularity, and the serum tumor marker CA-125 can be significantly elevated [4]. This combination often leads to a preoperative diagnosis of ovarian cancer, potentially resulting in over-treatment. We present a case from Afghanistan where this diagnostic dilemma was encountered, highlighting the need for awareness of this rare entity in diverse clinical settings.

## Case presentation

**Figure 1:**
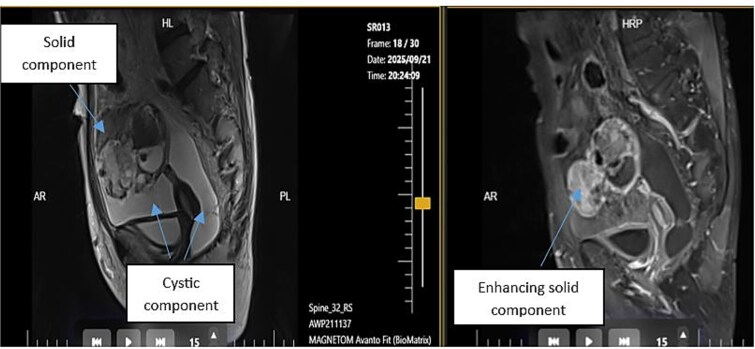
Pelvic MRI: (left) sagittal T2-weighted image showing a left adnexal complex cystic-solid mass; (right) fat-suppressed contrast-enhanced T1-weighted image showing enhancing solid component (arrow)—O-RADS 5.

A 53-year-old Afghan woman presented with abdominal swelling and pain. Ultrasound revealed a pelvic mass and severe ascites. CT showed a complex mass with cystic components and calcification, dimensions 88 × 65 × 21 mm, in favor of a tumoral lesion in the left ovary (O-RADS 5), accompanied by severe ascites due to presumed peritoneal seeding, suggestive of ovarian tumor with peritoneal carcinomatosis (Fig. 1). The uterus, right ovary, and other abdominopelvic areas were reported normal.

O-RADS 5 classification was assigned because of:(1) irregular solid tissue with contrast enhancement > signal of myometrium, (2) presence of ascites, and (3) peritoneal thickening mimicking implants.

Laboratory test results were CA-125 = 381 U/ml, TSH = 3.4 mIU/l, T4 = 14 pmol/l, hemoglobin = 7.5 g/dL, hematocrit = 23.3%; other tests were within normal limits.

**Figure 2:**
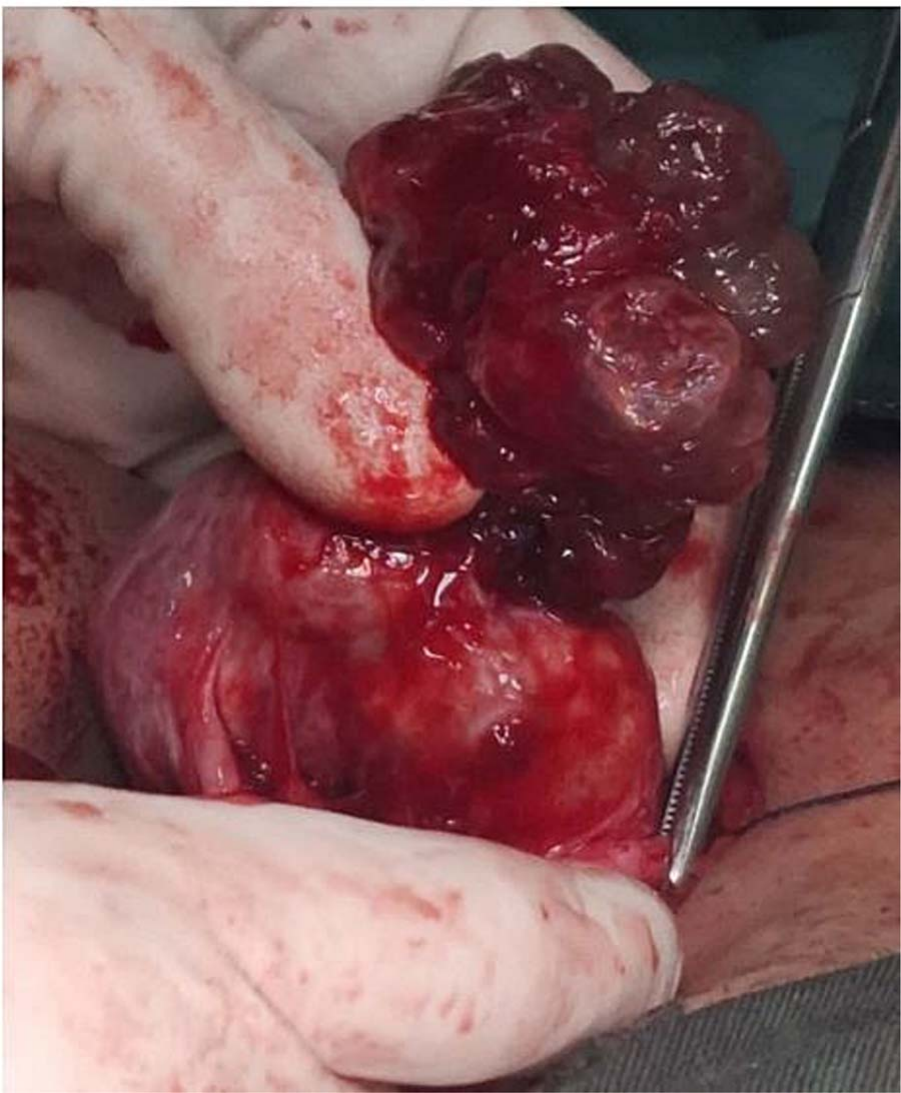
Intraoperative photograph of the resected left ovarian specimen revealing a solid-cystic cut surface with glistening appearance, consistent with benign *struma ovarii*.

The patient underwent drainage of 5 liters of ascites and laparoscopic biopsy. The biopsy result was reported as thyroid tissue compatible with *struma ovarii* (Fig. 2). Due to the discordance between strong radiologic suspicion (O-RADS 5) and limited biopsy sampling, the multidisciplinary tumor board concluded that sampling error could not be excluded. Consequently, empirical chemotherapy was initiated under the precautionary principle of ‘treating malignancy until proven otherwise.’ The patient was diagnosed with primary peritoneal carcinoma and received three cycles of chemotherapy with carboplatin and paclitaxel. Because the mass persisted and ascites increased, she received four more cycles (total seven cycles) and was referred for oncology consultation due to lack of response.

The patient was re-examined. MRI showed a multilocular cystic lesion with solid and fatty foci, measuring 100 × 80 mm, color score III (O-RADS 5). Severe ascites with multiple fibrotic septa but without nodularity was reported. No other lesions were seen.

### Pathology findings

#### Gross morphology and microscopic features

**Figure 3:**
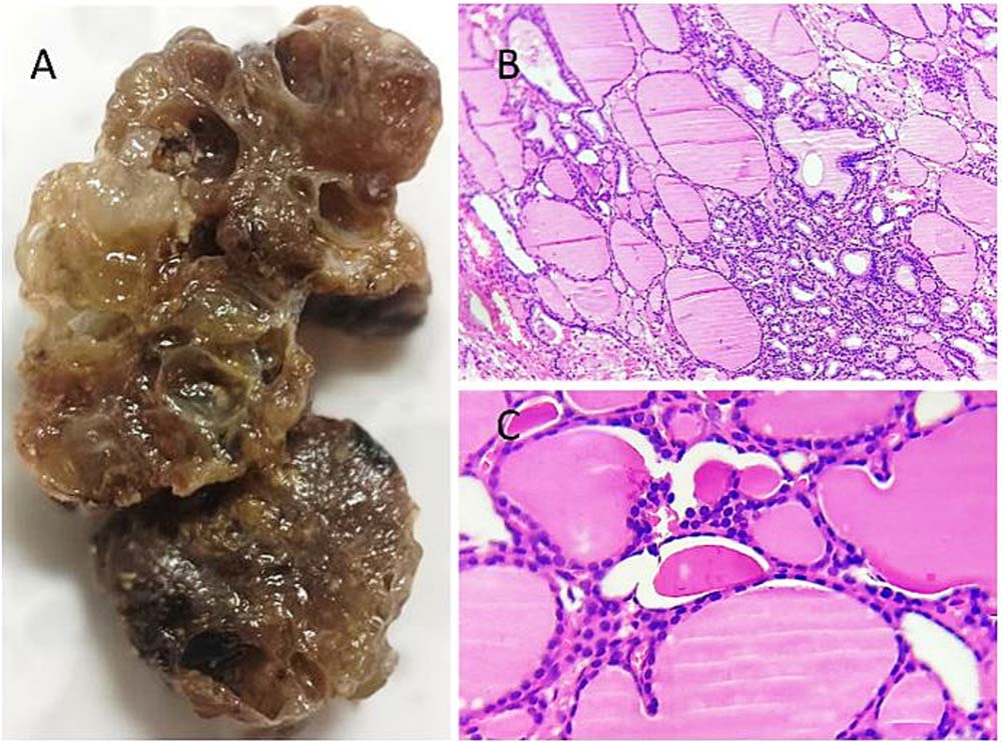
*Struma ovarii* pathology. (A) Ovarian tumor with solid-cystic and glistening cut surface. (B) Photomicrograph showing variably sized thyroid follicles lined by bland follicular cells (H&E, ×100). C: Higher power view (H&E, ×100)—no nuclear atypia or mitosis.

The resected left ovarian mass showed a solid-cystic cut surface with glistening appearance. Histopathological examination revealed variably sized thyroid follicles lined by bland follicular cells, with no nuclear atypia, no complex structures, and no mitosis to suggest malignancy (Fig. 3).

#### Immunohistochemical profile

IHC demonstrated PAX8 positive, TTF1 positive, and WT1 negative, confirming thyroid follicular origin.

#### Comparison with malignant differential diagnoses

The absence of high-grade cytologic features, capsular or vascular invasion, and extra-ovarian spread distinguished benign *struma ovarii* from malignant *struma ovarii* or metastatic thyroid carcinoma.

The mass, which preoperatively mimicked advanced ovarian cancer with its solid and cystic components, is shown after salpingo-oophorectomy. Final histopathology confirmed the diagnosis, explaining the patient’s chemo-resistant ascites and elevated CA-125. The postoperative course was uncomplicated. The patient was discharged on the third postoperative day. At follow-up several days later, she was asymptomatic, and repeat CA-125 had normalized to 17 U/ml. Table 1 summarizes the chronological diagnostic sequence.

**Table 1:** Chronologic diagnostic sequence.

Step	Intervention	Key findings	Rationale/clinical decision
**1**	CT imaging	Left adnexal mass, ascites, O-RADS 5	Suspicion of advanced ovarian cancer
**2**	Laparoscopic biopsy	Thyroid follicles (benign)	Discordant with radiology
**3**	Multidisciplinary tumor board	Sampling error possible	Empirical chemotherapy initiated
**4**	7 cycles carboplatin/paclitaxel	No response, persistent ascites	Treatment failure, re-evaluation
**5**	MRI	O-RADS 5, ascites, no nodules	Persistent suspicion of malignancy
**6**	Laparotomy + frozen section	Mature thyroid tissue, no malignancy	Definitive benign diagnosis
**7**	Final histopathology	Benign *struma ovarii*	Surgery curative

The patient’s general condition improved completely, and no recurrence or other problems were observed during 14 months of follow-up after surgery. She was counseled on the extremely low risk of recurrence, and long-term follow-up was advised.

## Discussion

This case illustrates a classic diagnostic pitfall in gynecologic oncology. The triad of a complex adnexal mass, severe ascites, and a significantly elevated CA-125 is highly suggestive of ovarian carcinoma [5]. However, as demonstrated, benign conditions like *struma ovarii* can present identically. Meigs syndrome is a rare phenomenon seen in approximately 1% of patients diagnosed with ovarian tumors [5–7].

The elevation of CA-125 is not specific to ovarian cancer. It can be elevated in various benign conditions, including endometriosis, pelvic inflammatory disease, and benign ovarian tumors. In *struma ovarii*, the mechanism is thought to be related to peritoneal irritation from the tumor or the ascitic fluid itself [4]. Ascites is found in 15%–30% of *struma ovarii* cases, further confounding the clinical picture [3].

Retrospective review of MRI revealed typical indicators of *struma ovarii* that were initially under-recognized: heterogeneous signal with hyperintense cystic components on T1-weighted imaging (due to colloid), iso- to hyperintense signal on T2-weighted imaging with a ‘stained-glass appearance,’ and vascular solid areas on fat-suppressed contrast-enhanced sequences. The absence of restricted diffusion was another suggestive clue. These features were initially overshadowed by the large size, solid nodularity, and ascites which biased classification toward malignancy.

According to O-RADS MRI criteria (version 2022), category 5 is assigned when an enhancing solid component with contrast enhancement > signal of myometrium, ascites, and peritoneal thickening are present. However, O-RADS lacks specificity for rare benign teratomas with thyroid tissue. Therefore, correlation with thyroid function tests and functional imaging (e.g. thyroid scintigraphy) is essential when *struma ovarii* is suspected.

The novelty lies in the prolonged misclassification as O-RADS 5 malignancy leading to chemotherapy before definitive surgery, reflecting the diagnostic challenge in resource-limited settings without intra-operative frozen section capability. The paper emphasizes interdisciplinary communication and awareness that benign *struma ovarii* can fully mimic advanced ovarian cancer both biochemically and radiologically.

Our case is particularly noteworthy due to the patient’s geographic and clinical context. In low -come region, where advanced diagnostic tools like MRI or PET-CT are not widely available, and intraoperative frozen section may be unreliable or absent, reliance on clinical judgment and basic imaging is high. This increases the risk of misdiagnosis and potentially unnecessary radical surgery, as was initially planned in some case. Typical indicators of *struma ovarii*, heterogeneous signal with hyperintense cystic components on T1 due to colloid, iso- to hyperintense on T2 with stained-glass appearance, and vascular solid areas, were retrospectively present but under-recognized. These details are now described with sequence names (T1-weighted, T2-weighted, fat-suppressed contrast-enhanced).

The literature confirms that *struma ovarii* frequently mimics ovarian malignancy. Yoo et al. reported that 40% of cases were preoperatively misdiagnosed as cancer [8]. Several case reports highlight pseudo-Meigs’ syndrome (ascites, pleural effusion) with elevated CA-125 as the primary driver of diagnostic error [4, 6, 9]. Erciyestepe et al. described a hormonally active *struma ovarii* in a 72-year-old woman presenting with a large multilobulated mass and ascites [7]. Liu et al. documented a giant *struma ovarii* with pseudo-Meigs’ syndrome [10]. Across all reports, the key lesson is identical: benign *struma ovarii* can clinically and radiologically mimic advanced ovarian cancer. Definitive diagnosis requires histopathology, and conservative surgery is curative in benign cases [1–5, 11].

A clinical analysis by Yang and Yu examines the diagnosis and treatment of 11 cases of *struma ovarii*, a rare, typically benign ovarian tumor composed of thyroid tissue, supplemented by a literature review. The study likely details the clinical and radiological presentation of these tumors, including the diagnostic challenges they pose, such as their potential to mimic ovarian malignancy through imaging or elevated tumor markers like CA-125, and emphasizes that a definitive diagnosis is achieved through postoperative histopathology. Their work reaffirms that the standard treatment is surgical, with the specific approach (cystectomy or oophorectomy) tailored to preserve fertility, when possible, based on patient age, tumor characteristics, and surgical findings [12].

The management of benign *struma ovarii* in a young woman wishing to preserve fertility is conservative surgery, such as cystectomy or oophorectomy [1–5]. In our patient, who was older and had completed her family, the performed surgery was ultimately adequate and because the uterus and ovaries were atrophic and normal, as well as the patient’s performance, there was no need for more extensive surgery.

### Limitation

Thyroid scintigraphy was unavailable locally at the time. Given the patient’s critical status and limited resources, management proceeded to chemotherapy without functional confirmation of the thyroid origin of the mass.

## Conclusion

This case from Afghanistan serves as a crucial reminder for clinicians worldwide, especially in resource-limited settings, to include *struma ovarii* in the differential diagnosis of a complex ovarian mass. While the clinical and serological features can be alarmingly similar to ovarian carcinoma, definitive diagnosis rests on histopathology. Efforts should be made to secure a frozen section intraoperatively, when possible, to guide surgical extent and prevent overtreatment. Clinicians and radiologists should recognize that *struma ovarii* may mimic ovarian malignancy even when CA-125 is markedly elevated. MRI features such as T1 hyperintense cystic components (due to colloid) and a ‘stained-glass’ appearance on T2-weighted imaging should raise suspicion for this benign entity, and these findings must always be correlated with thyroid function tests and, when feasible, thyroid scintigraphy. Furthermore, O-RADS criteria should be applied with clinical flexibility and multidisciplinary consultation whenever an unusual imaging pattern (solid plus cystic components with ascites) is encountered in a suspected benign teratoma.

## Data Availability

Data and materials are available upon request from the corresponding author.
